# Eclampsia and seasonal variation in the tropics - a study in Nigeria

**Published:** 2009-08-17

**Authors:** Ugochukwu Vincent Okafor, Efenae Russ Efetie, Obasi Ekumankama

**Affiliations:** 1 Department of Anaesthesia; 2 Department of Obstetrics and Gynaecology, National Hospital, Abuja, Nigeria

## Abstract

**Background:**

A retrospective observational study on the seasonal variation in the admission of eclampsia patients to the multi-disciplinary intensive care unit (ICU) of National Hospital, Abuja, Nigeria over a five-year span (March 2000 – March 2005) was carried out.

**Method:**

The patient’s case files and ICU records were used to extract the needed data. The diagnosis of eclampsia was based on clinical and laboratory findings by the obstetricians.

**Results:**

There were a total of 5,987 deliveries during the study period. Forty-six eclamptics were admitted to the ICU during the study period giving an ICU admission rate of 7.6/1000 deliveries. The average age of the patients was 28.6 years. Six patients (13%) were booked for antenatal care in the hospital, while forty patients (87%) were referred. Average duration of stay in the ICU was 4.6 days (range 1–42 days).

Thirty-one eclamptics (67.4%) were admitted to the ICU during the rainy season (April to October) and fifteen (32.6%) during the dry season (November to April). The rainy season is associated with a lower average high temperature and a higher humidity than the dry season. There is a view that holds that increasing humidity and a lower temperature is associated with increased incidence of eclampsia. There were thirteen deaths giving a case fatality rate of 28.2%. The causes of death were HELLP (haemolysis, elevated liver enzymes, low platelet count) syndrome in six patients, disseminated intravascular coagulation in two patients, and acute renal failure (ARF) in two patients. Septicemia, lobar pneumonia/heart failure and cerebrovascular accident accounted for one death each.

**Conclusion:**

In this study, we found an association between the rainy season and the incidence of eclampsia to our intensive care unit. This association should be further explored.

## Background

Eclampsia has been defined as the occurrence of convulsions not caused by coincidental neurologic disease, e.g. epilepsy in a woman whose condition also meets the criteria for pre-eclampsia [1]. Eclampsia remains a problem in the developing world despite improvements in antenatal care and facilities [2]. It is also a major cause of maternal mortality in Nigeria [3-7]. However, the pathogenesis of eclampsia and the events leading to it are poorly understood [8]. Amongst the factors being considered by researchers is the role of seasonal variation in its aetiology [9–11].

Nigeria has two distinct climatic zones; along the coast, the equatorial maritime air mass influences the climate, which is characterized by high humidity and rainfall. To the north, the tropical continental air mass brings dry dusty winds (harmattan) from the Sahara. Abuja, located in the middle belt region of Nigeria does not have the extreme climate of the coastal south and the arid parts of the upper north [12]. The main rains in Abuja occur between April and October. Average precipitation in Abuja ranges from zero inches in December, to 9.40 inches in September. The average high temperature is lower during the rainy season in Abuja [13].

The literature suggests there may be seasonal variation in the presentation of eclampsia, especially in the tropics. The aim of our study was to look for such a pattern among admissions to the intensive care unit of our hospital.

## Method

A retrospective observational study on the seasonal variation in admission of eclampsia patients to the multi-disciplinary intensive care unit (ICU) of National Hospital, Abuja, Nigeria over a 5 years span (March 2000 to March 2005) was carried out. National Hospital, Abuja is the apex tertiary hospital that serves the Federal Capital territory of Nigeria and some neighboring states.

The patients’ case files and ICU records were used to extract the needed data. The diagnosis of eclampsia was made based on clinical and laboratory findings by the obstetricians and admissions are effected after a review by the duty anaesthetist. The hospital protocol for the management of all eclampsia patients involves their admission to the intensive care unit for airway protection and higher medical care. The hospital does not have an intermediate care facility like a high dependency unit. The hospital has four consultant anaesthetists and nine consultant obstetricians.

The patients’ demographics, month of presentation, time of convulsions and maternal deaths were documented. The rainy season refer to the later part of the month of April, to October, and dry season from November to the early part of April of each year. Absence of daily temperature records were a limitation and depend on when the referred patients presented to our centre. From our experience it might be days after the first eclamptic fit as they might visit other centers before ours.

## Results

The number of total deliveries during the study period was 5,987 deliveries. Forty-six eclamptics were admitted to the ICU during the study period giving an ICU admission rate of 7.6/1000 deliveries.

The average age of the patients was 28.5 years (range 17 – 40 years). Table 1 I show the age distribution of the patients. Six patients were booked and forty were referred. Twenty-seven patients (58.7%) had antepartum eclampsia, twelve patients (26.1%) intrapartum eclampsia and six patients (1.3%) presented with postpartum eclampsia.

**Table 1:** Age distribution of eclamptics admitted to the ICU

**Patient’s age**	**Number**	**%**
17 – 20	5	11 (4.7)
21 – 25	8	17
26 – 30	12	26
31 – 35	16	35
36 – 40	5	11
é 40	-	-

Thirty-five patients (76.1%) delivered by caesarian section, while eleven (23.9%) delivered vaginally. All the eclamptics except two received oxygen enriched air via a Newport ventilator or nasal prongs/catheters.

Thirty-one eclamptics (67.4%) were admitted to the ICU during the rainy season (later part of April to October) and fifteen (32.6%) during the dry season (November to early part of April). Table 2 shows patient admission in relation to month of the year, and the monthly average for temperature and precipitation for 2003 and 2004. There were 13 maternal deaths giving a case fatality rate of 28.3%. The causes of death were HELLP (haemolysis, elevated liver enzymes, low platelet count) syndrome in six patients, disseminated intravascular coagulation in two patients, and acute renal failure (ARF) in two patients. Septicemia, lobar pneumonia/heart failure and cerebrovascular accident accounted for one death each.

**Table 2:** Number of eclamptics admitted to the intensive care unit during the months of 2000–2005

**Year**	**Month of the year**											
	1	2	3	4	5	6	7	8	9	10	11	12
2000			1							1		
2001	1	1							1			
2002		2		2		1	2		2	1	2	
2003				4	1		1	2		1	1	1
2004		1	2	2			4	2	3			2
2005	1												

## Discussion

Despite the reported reduced incidence in the Western World, eclampsia remains a significant cause of maternal mortality all over the world [14–16]. There was a preponderance of admissions to the ICU during the rainy season in this study. In an earlier study in Lagos, Nigeria, twenty-four years earlier, Agobe et al reported that the incidence of eclampsia varied significantly with the weather [11]. Another study in India supported this view [17]. The view holds that increasing humidity and a lower temperature is associated with increased incidence of eclampsia [11]. According to Agobe et al, protective action of arid conditions is consistent with the known effect of dehydration on convulsions of differing aetiologies and is attributable to increased pulmonary transpirational water loss. Eclampsia, exacerbated by cool humid conditions, may reflect the excessive water retention, due partly to suppressed pulmonary transpiration and partly to kidney malfunction in eclamptics.

A study on hypertensive patients shows that there is an association between blood pressure and the weather with lower daytime blood pressure and higher nighttime blood pressure during hot weather [18]. The lower blood pressure in hot weather has been attributed to increased vasodilatation and, loss of water and salt by sweating [19]. Cold weather is known to cause release of catecholamines, which increase blood pressure. The average high temperature was lower and humidity was higher during the rainy season in Abuja. Conversely, the low average temperature was higher during the rainy season. Our suspicion index was raised since any parturient that fits or is reported to have fitted is usually admitted to the ICU for observation and monitoring, and this centre is the only tertiary care institution in the federal capital territory of Abuja with a well equipped ICU. Besides, these are urban women with access to primary and secondary health care facilities.

A possible cause of the increase in presentation during the rainy season in this study may be due to malaria, which is transmitted more during the rainy season. A study in two West African countries showed that eclampsia was commoner in the rainy season [11, 20], with evidence of malarial parasites in the placenta of most eclamptics.  Another study in the Gambia, West Africa showed a 5.4 fold increase in the maternal mortality rate (MMR) due to eclampsia during the malaria season [21]. An epidemiological study in Zimbabwe, Southern Africa also showed a seasonal variation in the occurrence of eclampsia, similar to a prospective study done in Mozambique, also in Southern Africa [22, 23].

It is interesting that while the studies in sub-Saharan Africa show a relationship between seasons and occurrence of eclampsia, two studies in the United States concluded that the incidence of eclampsia was not influenced by climatic factors even in periods of high humidity [24, 25]. An interesting study in Norway concluded that there was a relationship between pre-eclampsia and seasons with a higher incidence during colder seasons [26]. The data in that study spanned a twenty-one year period. The study is important coming from a wealthy nation where access to health care services is not a problem (prenatal care is free), unlike studies from other nations with more diversity and poverty. The study emphasized the possible role of environmental factors like the diet during the seasons. That is similar to the possible role of environmental factors like malaria in this study. Another Scandinavian study from Sweden revealed that the prevalence of pre-eclampsia was reduced during the summer compared to the winter months [27]. Other studies from India and Ghana reported that more cases of eclampsia were seen during the rainy season [28, 29].

A lot of emphasis has been placed on good antenatal care and improved standard of living in most of the studies on eclampsia. Most of the women register for antenatal care in various hospitals and maternities, but are usually referred to us because of good ICU facilities.  A study in West Africa showed that despite access to maternal wards and essential obstetric care, many pregnant women still develop life-threatening complications including eclampsia [30]. However, due to the high incidence and mortality from eclampsia in Africa, it may be advisable to carry out further studies on the possible link between the seasons, malaria and eclampsia in the region.

## Conclusion

While eclampsia continues to take its toll on women, efforts are being made to understand its aetiology and possible prevention. Seasonal variations in the incidence may be one of them. This study shows an association between the rainy season and increased incidence of eclampsia. The role of malarial fever in this may need to be further investigated.

## Competing interest

This paper did not receive any funding from any source. The authors declare no conflict of interest

## Authors’ contribution

UVO and ERE collected data and analyzed them, while OO contributed to the editing of the paper.

## Acknowledgement

We thank the entire staff of the intensive care unit and records department of National hospital, Abuja for their cooperation during this study.
